# Supplementary material for: Improved Flame-Retardant and Ceramifiable Properties of EVA Composites by Combination of Ammonium Polyphosphate and Aluminum Hydroxide
Source: Polymers (Basel). 2019 Jan 12;11(1):125. doi: 10.3390/polym11010125 (PMC6401787; doi:10.3390/polym11010125)
Supplement: Supplementary file 1 [file polymers-11-00125-s001.pdf]

Supporting Information

Improved Flame Retardant and Ceramifiable Properties of EVA Composites by Combination of Ammonium Polyphosphate and Aluminum Hydroxide

Feipeng Lou[1], Kai Wu[1], Quan Wang[1], Zhongyu Qian[1], Shijuan Li[1] and Weihong Guo[1,*]

[1] Polymer Processing Laboratory, Key Laboratory for Preparation and Application of Ultrafine Materials of Ministry of Education, School of Materials Science and Engineering, East China University of Science and Technology, Shanghai 200237, P.R. China

* Correspondence: guoweihong@ecust.edu.cn; Tel.: 86-21-64251844

Table S1. CC data of EVA and EVA composites a flux of 50 kW m^{-2}.

Samples	PHRR (kW m^{-2})	THR (MJ m^{-2})	TTI (s)	Residue (wt%)	Peak SPR (1 ×10^{-2} m^2 s^{-1})
EVA1	217.4	63.7	64	59.3	2.2
EVA2	168.1	73.2	66	56.8	1.2
EVA5	178.9	58.3	73	61.3	2.4

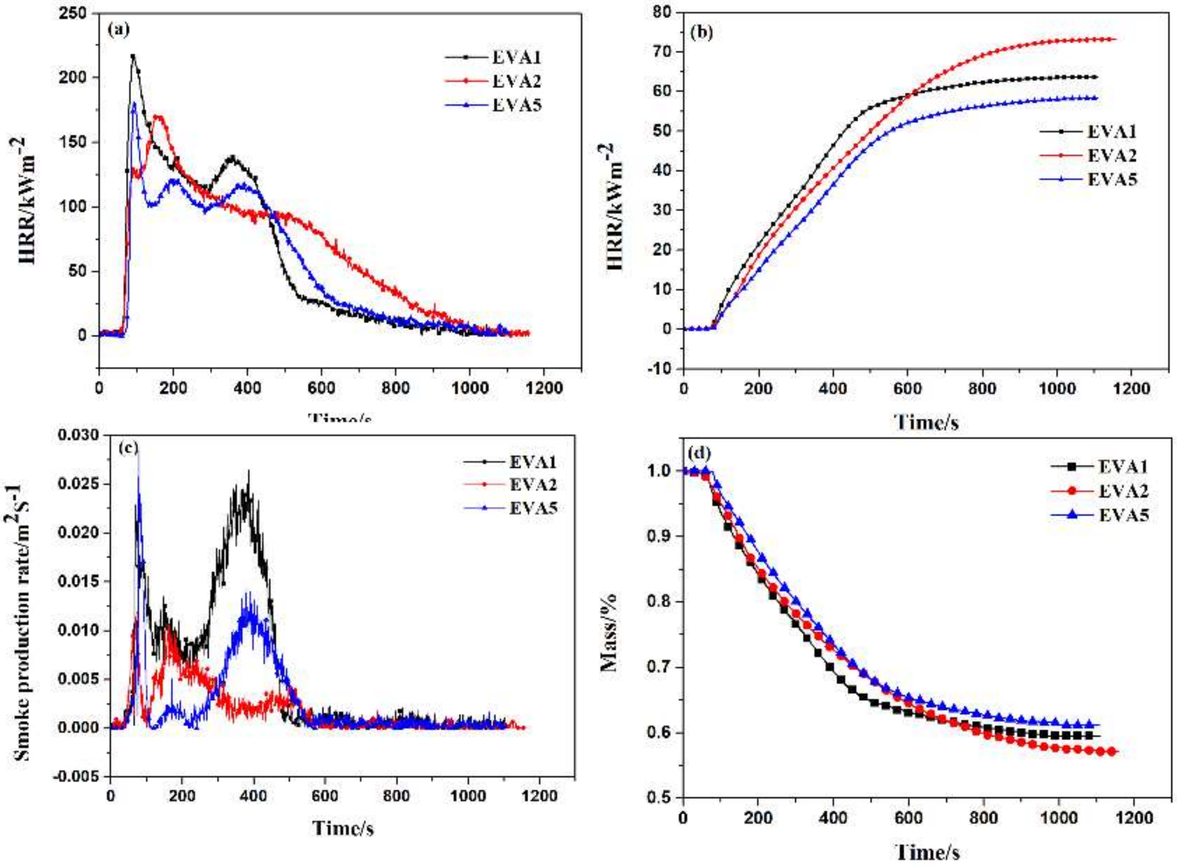

Figure S1. HRR (a), THR (b), SPR (c), and ML (d) curves of neat EVA and EVA composites at a flux of 50 kW m^{-2}.

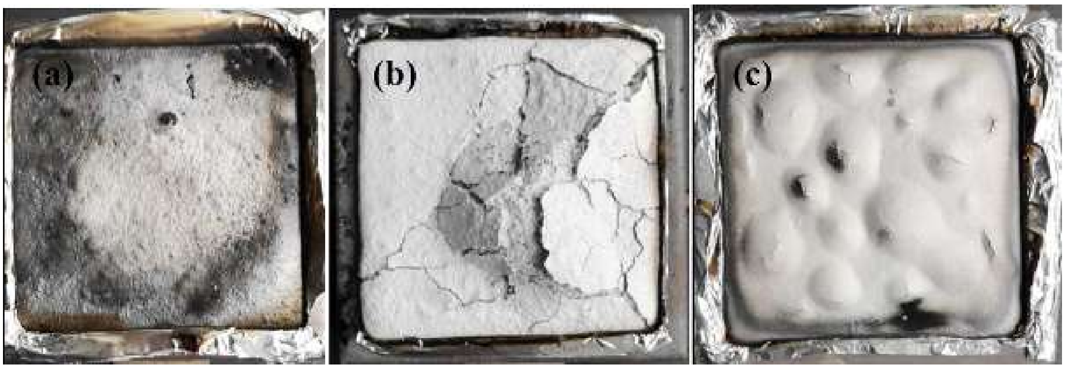

Figure S2. The digital photographs of the residues at a flux of 50 kW m^{-2}.

(a), EVA1; (b), EVA2; (c), EVA5;

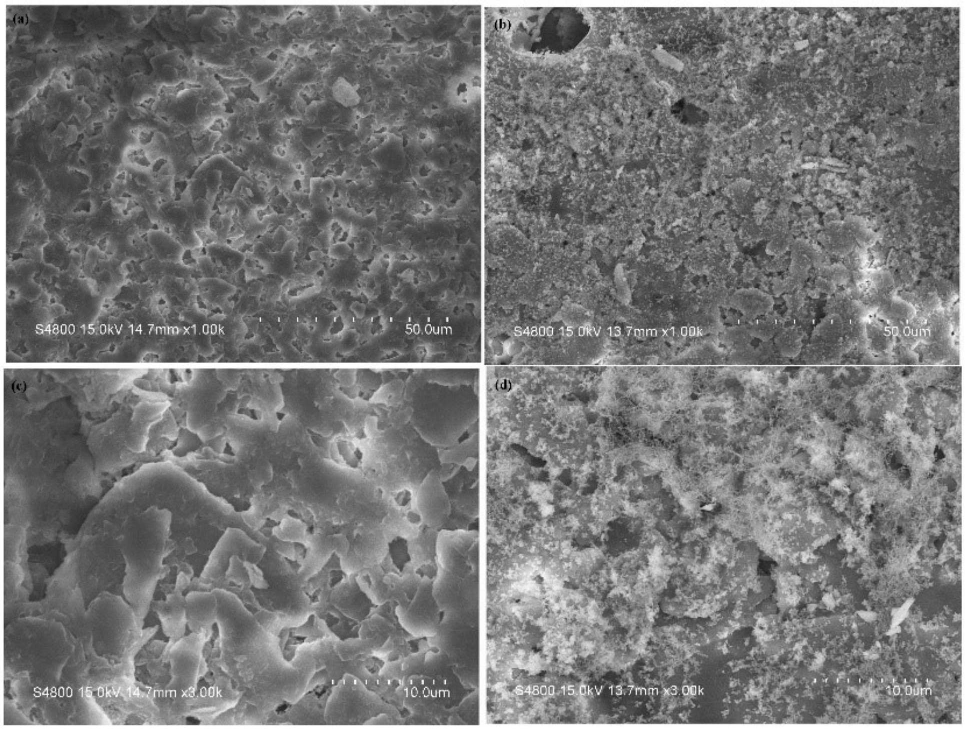

Figure S3. Surface morphologies of the residues after cone calorimetry. (a,c), EVA5; (b,d), EVA1;
